# Informational video on preoperative anxiety and postoperative satisfaction prior to elective cesarean delivery: a systematic review and meta-analysis

**DOI:** 10.1186/s40359-023-01499-3

**Published:** 2024-01-02

**Authors:** Mahsa Maghalian, Sakineh Mohammad-Alizadeh-Charandabi, Minoo Ranjbar, Farzaneh Aslanpour Alamdary, Mojgan Mirghafourvand

**Affiliations:** 1 Department of Midwifery, Faculty of Nursing and Midwifery, Tabriz University of Medical Sciences, Tabriz, Iran; 2 Department of Physiology, Shahid Beheshti University of Medical Sciences, Tabriz, Iran; 3 Social determinants of Health Research Center, Faculty of Nursing and Midwifery, Tabriz University of Medical Sciences, Tabriz, Iran

**Keywords:** Cesarean, Birth, Anxiety, Video, Post-operative satisfaction, Meta-regression

## Abstract

**Background:**

Preoperative anxiety affects 60 to 80% of patients who are candidates for surgery. Reducing preoperative anxiety can improve surgical outcomes, shorten hospital stays, and minimize disruptions in lifestyle. Having information affects people ability to identify important points and improve their understanding, and lack of information causes fear and anxiety, which negatively affects decision-making. Studies have shown that the intervention of education before cesarean section has a beneficial effect on women anxiety level. Providing information before surgery can reduce patients’ anxiety. This study was conducted to determine the effect of information video before elective cesarean delivery on preoperative anxiety and post-operative satisfaction.

**Methods:**

The search for relevant studies was systematically conducted in PubMed, Scopus, Web of Science, Cochrane Library, SID (Persian database), and Google Scholar (search engine) until July 4, 2023, in both English and Persian languages. The revised tool for assessing the risk of bias in randomized trials (RoB 2.0) and ROBIN-I were used to evaluate the risk of bias, and heterogeneity was assessed using I². In cases of high heterogeneity, a random effects model was used instead of a fixed effects model. Subgroup analysis was performed based on the duration of the video, and the type of intervention for the primary outcome. Sensitivity analysis was conducted based on the type of study. A random-effects meta-regression analysis was conducted to identify potential sources of high heterogeneity for preoperative anxiety. The certainty of the evidence was assessed using GRADE.

**Results:**

A total number of 557 articles were found in databases. Three hundred sixty-eight studies were screened based on their titles, abstracts, and full texts. Of these, 16 studies were assessed for eligibility, and 7 were excluded. Ultimately, nine papers were included. Meta-analysis results showed that the information video before elective cesarean delivery compared to control group may have little or no effect on preoperative anxiety, but the evidence is uncertain (SMD − 0.22, 95% CI -0.51 to 0.06, 9 trials, 1020 participants, I^2^ = 80%; very low-certainty evidence). Also, it probably increases the post-operative satisfaction (SMD 0.26, 95% CI 0.10 to 0.42, 5 trials, 618 participants, I^2^ = 0%; Moderate-certainty evidence). The random effect meta-regression analyses indicated a significant correlation between the mean age of the intervention group (β = 0.137, P < 0.001) and the mean age of the control group (β = 0.150, P = 0.0246) with effect size.

**Conclusion:**

This study found that watching an informational video prior to elective cesarean delivery resulted in a decrease in preoperative anxiety. However, it is important to note that the reduction was not statistically significant, and there was a high level of inconsistency among the results. Nonetheless, the intervention did lead to an improvement in women’s post-operative satisfaction. To determine the optimal time duration and content type of informational videos, further studies with more appropriate methodology are necessary.

**Supplementary Information:**

The online version contains supplementary material available at 10.1186/s40359-023-01499-3.

## Introduction

Giving birth is an important life event for a woman and she may experience various emotions, including anxiety [1]. One of the most common surgical procedures in the field of obstetrics and gynecology worldwide is cesarean Sect. [2]. The use of cesarean section has increased over the past three decades, with the global average rate of cesarean section rising from 6.7% in 1990 to 18.6% in 2014 and 21% in 2015 [3]. Currently, about one-third of births in many developed countries are delivered via cesarean Sect [4]. Cesarean section is a major surgical operation that carries risks for the health of both mothers and babies. Compared to vaginal delivery, cesarean delivery without medical indication is more likely to be associated with a range of maternal complications, including maternal mortality, infection, bleeding, adhesions, rupture, bleeding in subsequent pregnancies, prolonged hospital stay, and/or recovery time, and drug reactions. Additionally, babies born by cesarean section are at an increased risk of experiencing breathing problems, respiratory distress, low Apgar scores, fetal harm, allergic rhinitis, food allergies, asthma, and childhood-onset type 1 diabetes [5]. In addition to physical problems, the cesarean Sect. [6] can also have psychological consequences. These may include depression, anxiety, post-traumatic stress, health-related quality of life issues, problems with infant feeding, satisfaction, and self-esteem [7].

Preoperative anxiety leads to an increase in heart rate, blood pressure, and cardiac excitability, resulting in arrhythmia. The extent and duration of anxiety can lead to reduced wound healing, increased risk of infection, increased postoperative pain, and increased demand for analgesics [8, 9]. Maternal anxiety can also lead to a negative perception of pain and a decrease in breastfeeding [9]. Negative birth experiences have long-term effects on several aspects of a patient’s life, such as an increased risk of postpartum depression [10]. Postpartum depression is a condition that can potentially develop into a chronic health issue, leading to significant costs for both families and society [11]. Additionally, cesarean delivery can impact women’s future fertility decisions. A study conducted by Halla et al. (2016) found that mothers who underwent a cesarean section were less inclined to have additional children. This trend was attributed to postpartum psychological problems as a potential explanation [12].

Anxiety has been described in two forms: state anxiety and trait anxiety [9]. State anxiety is a temporary condition in an individual’s emotional life that includes mental sensations, tension, unease, nervousness, worry, and activation of their autonomous nervous system. Trait anxiety refers to relatively stable individual differences in the tendency to perceive threatening or dangerous stressors and react to such situations with increasing frequency and severity in state anxiety [13]. Preoperative anxiety affects 60 to 80% of patients who are candidates for surgery [14]. Reducing preoperative anxiety can improve surgical outcomes, shorten hospital stays, and minimize disruptions in lifestyle [15]. Providing information before surgery can reduce patients’ anxiety [16]. Given the high prevalence of preoperative anxiety, there have been extensive evaluations of various treatment options, including both pharmacological and non-pharmacological approaches. Non-pharmacological interventions have garnered particular attention due to the potential side effects associated with drug treatments. Some of the non-pharmacological methods that have been explored include cognitive-behavioral therapy, music therapy, preoperative preparation videos, aromatherapy, hypnosis, guided imagery relaxation therapy, and massage [17].

There are two main categories of methods for reducing anxiety: pharmaceutical and non-pharmaceutical. Non-pharmaceutical methods include pre-treatment counseling, effective communication, systemic desensitization, and hypnosis. Additionally, studies have demonstrated that aromatherapy, music therapy, and acupuncture are effective in controlling patient anxiety [18]. The ideal method for presenting information is unknown, and while written information has been utilized effectively as a means of providing information to patients, not all patients possess sufficient literacy skills to read and understand informational materials. [19]. The mechanism of pre-cesarean training is related to the interaction between situational anxiety, information retention, and memory [17].

Herman and Kreuzer found in their research that video films are useful tools alongside routine care [20]. Teaching mothers through video-based education helps develop and improve their skills and knowledge, resulting in better care. Online education and educational modules using video as a novel method for providing continuous education have emerged [21]. Video-based education strengthens mothers’ learning and can present material in ways that verbal descriptions or speech alone cannot [22]. Given the beneficial effects of educational multimedia on reducing patient anxiety and the lack of evidence-based studies on this topic before cesarean surgery, this study was conducted.

### Objective

To examine the effect of information video before elective cesarean delivery on preoperative anxiety and operative satisfaction.

## Methods

To conduct this meta-analysis, the PRISMA statement was followed [23], and the study protocol was registered in PROSPERO (International Prospective Register of Systematic Reviews) on February 17, 2023, under the registration code CRD42023395924, prior to the start of the study.

### Eligibility criteria

We conducted a study that included all quasi-experimental (non-randomized) and randomized controlled trials (RCTs) published in English or Persian languages, which investigated the effect of preoperative information video (regardless of the intervention duration) on preoperative anxiety (primary outcome) and operative satisfaction (secondary outcome) among women undergoing planned cesarean section. The details of inclusion and exclusion criteria based on PICOS are presented in Table 1.

**Table 1:** Inclusion and exclusion criteria

Category	Inclusion criteria	Exclusion criteria
Population	Women undergoing planned cesarean section	Women undergoing emergency cesarean section
Intervention	Any preoperative information video related to various types of anesthesia (such as spinal or general anesthesia), cesarean delivery, the hospital environment, and related care was included, regardless of the intervention duration or content. This approach was undertaken to capture a broad range of approaches in preoperative informational videos	Other interventions except information video included structured education, leaflets, oral briefings, and handouts
Outcome	Preoperative anxiety and post-operative satisfaction as outcomes	Studies that did not assess preoperative anxiety or post-operative satisfaction
Study design	All quasi-experimental (non-randomized) and RCTs	Observational studies, conference abstracts, letters, and reviews
Other	Until July 4, 2023, there were no time restrictions for the inclusion of studies The studies published in English or Persian languages were included to provide access to a wide range of literature and data sources. Moreover, the research team’s proficiency in both languages enables efficient analysis and interpretation of the findings from these studies	None

RCT = Randomized controlled trial

### Search methods

The search for relevant studies was conducted systematically in several databases, including PubMed, Scopus, Web of Science, Cochrane Library, SID (Persian database), and Google Scholar (search engine), until Jul 04, 2023. In addition, the references of the identified articles were searched, and manual searching was performed to find more relevant studies. The keywords used as both free and Mesh terms in the databases are listed below.

((labor) OR (peripartum) OR (childbirth) OR (cesarean) OR (elective cesarean) OR (C-section) OR (c-section) OR Cesarean Section [MeSH] OR Elective Surgical Procedures*) AND (Stress OR Anxiety OR Panic OR psychology OR surgery OR surgical OR preoperative OR “preoperative anxiety” OR Preoperative Care) AND “Informative* Video” OR Media OR audio video OR recording, videotape OR “multimedia” OR “Patient Education” OR virtual reality AND (“randomized-controlled trial’’ OR “controlled clinical trial” OR randomized OR randomly OR trial OR RCT).

The search strategy for each database is provided in the additional file.

### Selection of studies

Using pre-defined inclusion and exclusion criteria, two authors (MMa, MR) independently reviewed the studies obtained from the search based on their titles and abstracts, and when necessary, the full text. If any discrepancies arose between the two authors, a third author (MMi) was consulted to resolve them.

### Data extraction

Using a data extraction form that included information such as author name and publication year, country, sample size, participant age, BMI (Body Mass Index), Gestational age at delivery, and the number (percent) of nulliparous women, intervention, comparison group, duration of intervention, outcomes, outcome measurement tool, and results, two authors (MMa, MR) independently extracted data from the included studies for the meta-analysis. Furthermore, if needed, the authors corresponded with the authors of the included studies via email.

### Assessment of risk of bias in included studies

Two authors (MMa, MR) evaluated the risk of bias for each of the included studies independently, using the revised tool for assessing the risk of bias in randomized trials (RoB 2.0) [24]. They assessed the domains of the randomization process, deviations from the intended interventions, missing outcome data, measurement of the outcome, and selection of the reported result, and classified them as low risk, high risk, or some concerns. For Non-randomized studies, the ROBINS-1 tool was used to evaluate the risk of bias. If there was any disagreement between the two authors, a third author (MMi) was consulted.

### Certainty of evidence

The certainty of evidence was assessed using the Grading of Recommendations Assessment, Development, and Evaluation [25] framework, which classifies evidence into four categories: high, moderate, low, and very low. This assessment includes five items: risk of bias, imprecision, inconsistency, indirectness, and publication bias [26].

### Data analysis

The meta-analysis was performed using RevMan version 5.3 software. For continuous outcomes, the standardized mean difference with a 95% confidence interval was used when different tools for outcome measurement were employed in studies. For outcomes reported both before and after the intervention, mean difference and standard deviation (SD) difference were estimated using the recommended methods in the Cochrane Handbook for Systematic Reviews of Interventions [27]. The mean and SD for preoperative anxiety scores before and after the intervention for both intervention and control groups are given in an additional file, Table 1. In cases where only the interquartile range was reported, SD was estimated by multiplying it by 1.35 and using the methods mentioned in the Cochrane Handbook for Systematic Reviews of Interventions [27, 28].

According to the recommendations of Cochran’s handbook, the interpretation of I² values is as follows: I² values between 0% and 40% may not be considered important, while values between 30% and 60% suggest moderate heterogeneity. Therefore, in the current study, an I² statistic greater than 30% is considered as high heterogeneity. For these cases, a random effects model is used instead of a fixed effects model [27, 29]. Subgroup analysis was performed based on the duration of the video (post hoc) and the type of intervention for the primary outcome. Since the number of studies included was less than 10, publication bias was not assessed.

A random-effects meta-regression analysis was conducted using Comprehensive Meta-Analysis V3 to identify confounder factors and potential sources of high heterogeneity for preoperative anxiety (primary outcome). These sources included the mean age of the intervention group, the mean age of the control group, the total sample size, baseline severity [30], year of publication, the percentage of participants with above-high education, type of outcome measurement tool, percentage of nulliparous women [31], and the duration of the video [32, 33].

### Sensitivity analysis

Sensitivity analysis was performed based on the type of study, and non-randomized studies were excluded due to concerns regarding potential methodological bias and heterogeneity.

## Results

### Results of the search

**Fig. 1:**
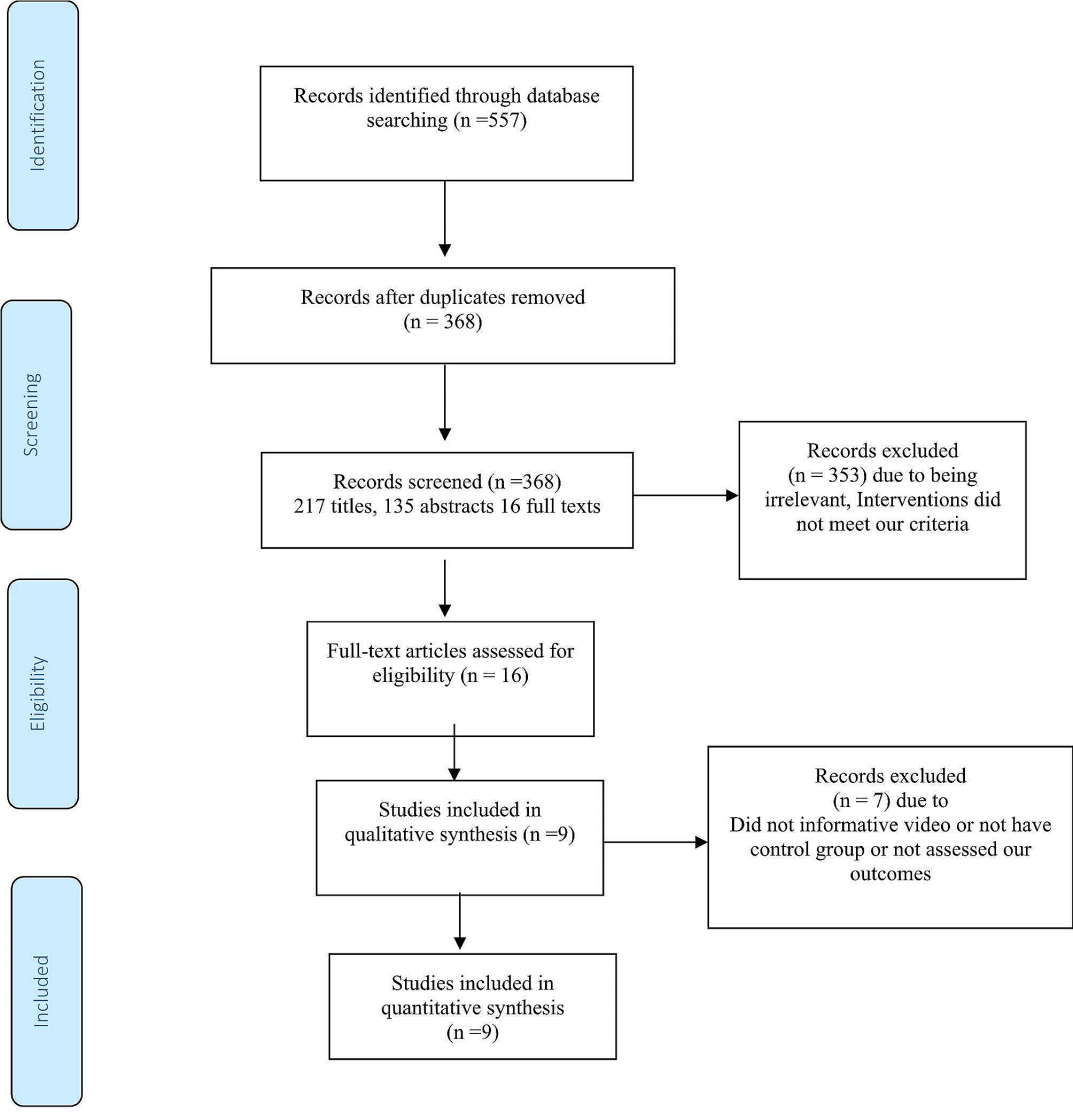
Flow diagram of the systematic literature search

A systematic search of PubMed, Scopus, Web of Science, SID, and The Cochrane Library databases yielded 557 records. After removing duplicates, 368 studies were screened based on their titles, abstracts, and full texts. Of these, 16 studies were assessed for eligibility, and 7 were excluded due to emergency cesarean Sect. [34], lack of a control group [35], not performing the intended intervention (information video) [36, 37], not matching the type of study with our criteria [38, 39], or not assessing the outcomes of interest [40]. Ultimately, nine papers were included, comprising eight randomized controlled trials (RCTs) and one non-RCT. (Fig. 1)

### Study characteristics

The characteristics of the studies included in the present systematic review and meta-analysis are presented in Table 2. The total sample size of the included studies was 1020 women, with the largest sample size being 160 in the study by Rabiei et al. [41] and 175 in the study by Purcell-Jones et al. [42], while the smallest sample size was 37 in the study by Kanyeki et al. [43]. The studies were conducted in 9 different countries, including West Indies [44], Iran [41], Turkey [45], South Africa [42], China [46], Netherlands [47], Israel [48], Australia [49] and East Africa [43], and were conducted between 2013 and 2023. Most of the studies were conducted in the last 4 years (2019–2023), with the study by Rabiei et al. [41] conducted in 2017 and the study by Eley et al. [49] conducted in 2013. All studies were RCTs, except for one study [42], which was non-RCT.

**Table 2:** Characteristics of included studies

First Author/date of publication / Country	Type of clinical trial	Sample size	Age of participants (Years)	BMI (kg/m2)	Gestational age at delivery (Weeks)	Number (Percent) of nulliparous women	Intervention	Comparison	Outcomes	Outcome measurement	Results
Kanyeki et al. 2022/ East Africa	RCT	Video group: 21 Control group: 16	35.2 ± 6.12	NR	NR	3.0 (14.3)	The video was a 7.55-minute film that featured actors who were residents and theatre staff. It was filmed in the university hospital theatres with the intention of creating a sense of familiarity for patients. The video underwent careful editing to include a running commentary of the events, providing an overview of common risks and anticipated outcomes of a successful block. It specifically focused on addressing the typical concerns expressed by patients regarding spinal anesthesia, which were identified through a comprehensive literature review in addition to the standard pre-anesthetic review	Standard pre anaesthetic review	Preoperative anxiety	STAI	There was no statistically significant difference in change of anxiety score between the video and control groups
Eley et al. 2013/ Australia	RCT	Video group: 52 Control group: 58	31.1 ± 5.0	26.1 ± 4.9	39.0 ± 0.8	15.0 (29.0)	The video lasted four and a half minutes and effectively utilized actors and a narrative to portray a typical patient journey. It began with the administration of ranitidine in the holding bay and concluded with the patient’s arrival in the post-anesthetic care unit. The primary objective of the video was not to provide detailed information about the surgical process. Instead, it employed animations to explain the basic anatomy of neuraxial anesthesia. The video featured explanations and demonstrations of various aspects, including the insertion of the intravenous cannula, patient positioning for neuraxial anesthesia, insertion of the neuraxial anesthetic, and the use of patient monitoring. Additionally, it offered descriptions of common sensations experienced after neuraxial anesthesia for a cesarean section. However, the video did not address the risks and side effects associated with neuraxial anesthesia, as its main focus was to showcase the usual care process	underwent usual care only	Preoperative anxiety Post-operative satisfaction	STAI MSSCS	There was statistically significant difference in change of anxiety score between the video and control groups There was no statistically significant difference in change of satisfaction score between the video and control groups
Miremberg et al. 2022/ Israel	RCT	Video group: 64 Control group: 68	30.±6.1	27.3 ± 4.6	38.5 ± 0.8	41(64.1)	A 4-minute video was created to encompass real-life images that portrayed all aspects of a cesarean delivery. It comprehensively covered the entire process, starting from the patient’s admission to the hospital and introducing the medical staff they are likely to encounter, such as midwives, anesthesiologists, and obstetricians. The video showcased various procedures, including intravenous line insertions, preoperative medical treatment, and scenes from the operating room and postoperative recovery room until the patient’s discharge from the hospital. To enhance understanding, animations were incorporated to demonstrate neuraxial anesthesia and illustrate the location of the surgical incision. The information presented in the video was based on the expertise of healthcare providers who aimed to address frequently asked questions by mothers. By offering both routine medical service and educational support, the video sought to provide comprehensive assistance	Received standard of care	preoperative anxiety Post-operative satisfaction	STAI 4-point Likert scale	There was statistically significant difference in change of anxiety score between the video and control groups There was no statistically significant difference in change of satisfaction score between the video and control groups
Noben et al. 2019/ Netherlands	RCT	Video group: 49 Control group: 48 97	32.6 ± 3.6	25.6 ± 4.8	39.0 ± 0.7	25(51)	The 4.45-minute 360° virtual reality video offered a comprehensive perspective on various aspects of a cesarean delivery, providing viewers with a detailed understanding of the entire process. It commenced with the patient’s admission to the ward and seamlessly transitioned to scenes within the operating room. The video effectively showcased the precise placement of spinal analgesia and captured the poignant moment when the gynecologist lifted the baby above the sterile environment during birth. It is worth noting that the video adhered to the highest standard of care. However, it deliberately abstained from including any surgical content that would explicitly show the specific area of incision	Received standard of care	Preoperative anxiety	VAS	There was no statistically significant difference in change of anxiety score between the video and control groups There was no statistically significant difference in change of satisfaction score between the video and control groups
Che et al. 2020/ China	RCT	Video group: 61 Control group: 60 121	27.8 ± 4.3	29.9 ± 3.9	39.03 ± 0.6	37(61)	The 8.5-minute video provided a real-life depiction of the process of administering elective cesarean anesthesia. It followed expectant mothers from preoperative visits with anesthesiologists to the operating room and highlighted the responsibilities of anesthesiologists in ensuring safety. The video showcased nurses opening intravenous channels, anesthesiologists administering anesthesia, and the subsequent return to the ward after the cesarean section. It emphasized the use of equipment and rescue medicine and addressed important aspects such as preoperative and intraoperative rights of mothers and the after-effects of anesthesia. Notably, the video did not include information about the risks of intraspinal anesthesia, surgical procedure details, or graphic images	Received standard of care	Preoperative anxiety, Post-operative satisfaction	STAI MSSCS	There was statistically significant difference in change of anxiety score between the video and control groups There was statistically significant difference in change of satisfaction score between the video and control groups
Yilmaz et al. 2019/ Turkey	RCT	Video group: 55 Control group: 51 106	31.2 ± 5.3	31.5 ± 5.8	NR	NR	The patients received multimedia education by watching a 4-minute and 15-second information video about general anesthesia on a personal computer equipped with headphones. The video offered a comprehensive explanation of the procedure, including detailed information about the associated risks and benefits. Furthermore, an anesthesiologist accompanied the patients and provided brief verbal information in conjunction with the video	In addition to the Solely brief verbal information	Preoperative anxiety Post-operative satisfaction	STAI 5-point Likert scale	There was statistically significant difference in change of satisfaction score between the video and control groups There was statistically significant difference in change of satisfaction score between the video and control groups
Purcell-Jones et al. 2019/ South Africa	Non-RCT	Video group: 83 Control group: 92 175	31.5 ± 5.2	NR	NR	NR	The video had a duration of 3 min and portrayed the experience of a Xhosa woman undergoing a spinal anesthetic for her cesarean delivery. The narration in the video was primarily conducted by a Xhosa-speaking nurse. Additionally, a segment of the video featured the patient herself describing her personal experience with the spinal anesthesia. This was incorporated alongside verbal explanations of the spinal anesthesia procedure	“They received the “usual care” verbal explanations of their spinal anesthesia”	Preoperative anxiety Post-operative satisfaction	NVAAS MSSCS	There was statistically significant difference in change of anxiety score between the video and control groups There was no statistically significant difference in change of anxiety score between the video and control groups
Rabiei et al. 2017/ Iran	RCT	Video group: 81 Control group: 81 162	27.8 ± 4.54	23.9 ± 4.4	38.6 ± 2.2	39(48)	The intervention group was provided with a 12-minute video created by the research team, which they watched on the day prior to their operation. This video encompassed detailed explanations about the surgical procedure, various types of anesthesia, essential pre- and post-operative care requirements, as well as information about the hospital environment. At the Persian Gulf Hospital in Bushehr, it was a standard procedure to offer this video to patients the night before their operation, in addition to providing regular care	No intervention	Preoperative anxiety	APAIS	There was statistically significant difference in change of anxiety score between the video and control groups
Singh et al. 2023/ West Indies	RCT	Video group: 40 Control group: 40 80	32.0 ± 6.14	NR	NR	NR	The ten-minute educational anesthetic video offered a comprehensive overview of a patient’s journey, starting from their arrival at the operating theatre and concluding with their discharge from the recovery room. It encompassed various essential topics, including the operating theatre environment, the neuraxial technique, patient monitoring throughout the surgical procedure, the correct positioning for the neuraxial block, the advantages associated with a neuraxial block, potential complications, and the perioperative period leading up to discharge. Notably, the video incorporated real-life images of a patient undergoing a spinal anesthetic for a cesarean section, emphasizing that proper informed consent was obtained	No educational video	Preoperative anxiety Post-operative satisfaction	STAI MSSCS	There was statistically significant difference in change of anxiety and post-operative satisfaction score between the video and control groups

RCT = Randomized controlled trial; STAI = The State-Trait Anxiety Inventory (score 20 to 80); VAS = visual analog scale (score 0 to 10); MSSCS = Maternal. Satisfaction **Scale** for Caesarean Section (score 22 to 154); NVAAS = Numerical Visual Analog Anxiety Scale (score 0 to 100); APAIS = The Amsterdam Preoperative Anxiety and Information Scale (score 6 to 30); BMI = body mass index

All included studies reported preoperative anxiety as an outcome measure, and among them, five studies [42, 44–46, 48] also assessed postoperative satisfaction. The intervention in all studies involved watching a preoperative educational video on the day of the cesarean section. In five studies, the video focused solely on anesthesia, while in four studies [41, 45, 47, 48], the video provided information on both anesthesia and the cesarean section procedure. The duration of the educational videos ranged from 3 min [42] to 12 min [41].

The participants were women aged 16 years and older without a history of psychiatric or anxiety disorders, but in one study [47], women with a history of depression or anxiety were included, although the number of such women in each group was small. In 7 studies, women with a history of previous Cesarean section or anesthesia were included [41–44, 46, 47, 49], while in 2 studies, women did not have such a history [45, 48]. Pregnancy age at the time of Cesarean section was reported in 6 studies, and all participants were at or above 37 weeks of gestation.

In the included studies, there was no significant difference in the level of education between the intervention and control groups, except in one study [47] where participants in the control group had a higher literacy level. Additionally, in one study [45], there was no mention of the participant’s educational level in the baseline information.

In one study [47], the visual analog scale (VAS) for anxiety was used to measure preoperative anxiety, which is a 0–10 cm scale, with 0 indicating no anxiety and 10 indicating the highest level of anxiety. In another study [42], the Numerical Visual Analog Anxiety Scale (NVAAS) was used, which measures anxiety levels on a 0-100 mm scale [50]. In one study [41], The Amsterdam Preoperative Anxiety and Information Scale (APAIS) was used to measure anxiety. It consists of two sections: preoperative anxiety, which is assessed using four questions, and the need for preoperative information, which is assessed using two questions. The scale is scored on a 5-point Likert scale, with a minimum score of 6 and a maximum score of 30. The responses range from 1 (“not at all”) to 5 (“extremely”) [51]. In 6 studies [43–46, 48, 49], the State-Trait Anxiety Inventory (STAI) was used, which is a 40-item tool that measures two types of anxiety: state anxiety (STAI-S) and trait anxiety (STAI-T). It is scored on a 1–4 Likert scale, with a minimum score of 20 and a maximum score of 80 for anxiety [52].

Operative satisfaction was assessed in 4 studies [42, 44, 46, 49] using the maternal satisfaction scale for cesarean section (MSSCS). This tool consists of 22 questions and a 7-point Likert scale, with a minimum score of 22 and a maximum score of 154 [53]. In one study, a 5-point Likert scale was used to assess satisfaction.

### Assessment of risk of bias

Four studies [43, 44, 46, 49] showed a low risk of bias in the randomization process. Four studies [41, 45, 47, 48, 54] were rated as some concern due to insufficient information regarding allocation sequence random, allocation sequence concealment, and baseline balance. The intended intervention bias was low risk in all the studies except one study that was considered as high risk due to there being no blinding of participants and no information about the statistical analysis used to estimate the effect of assignments on intervention and its substantial impact on the result.

**Fig. 2:**
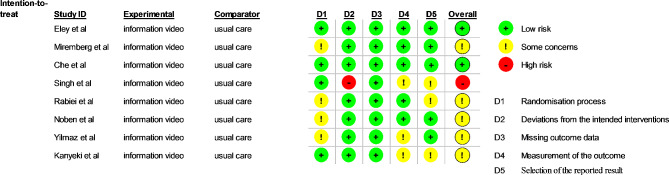
Risk of bias graph. Review authors’ judgments about each risk of bias item presented as percentages across all included studies

**Fig. 3:**
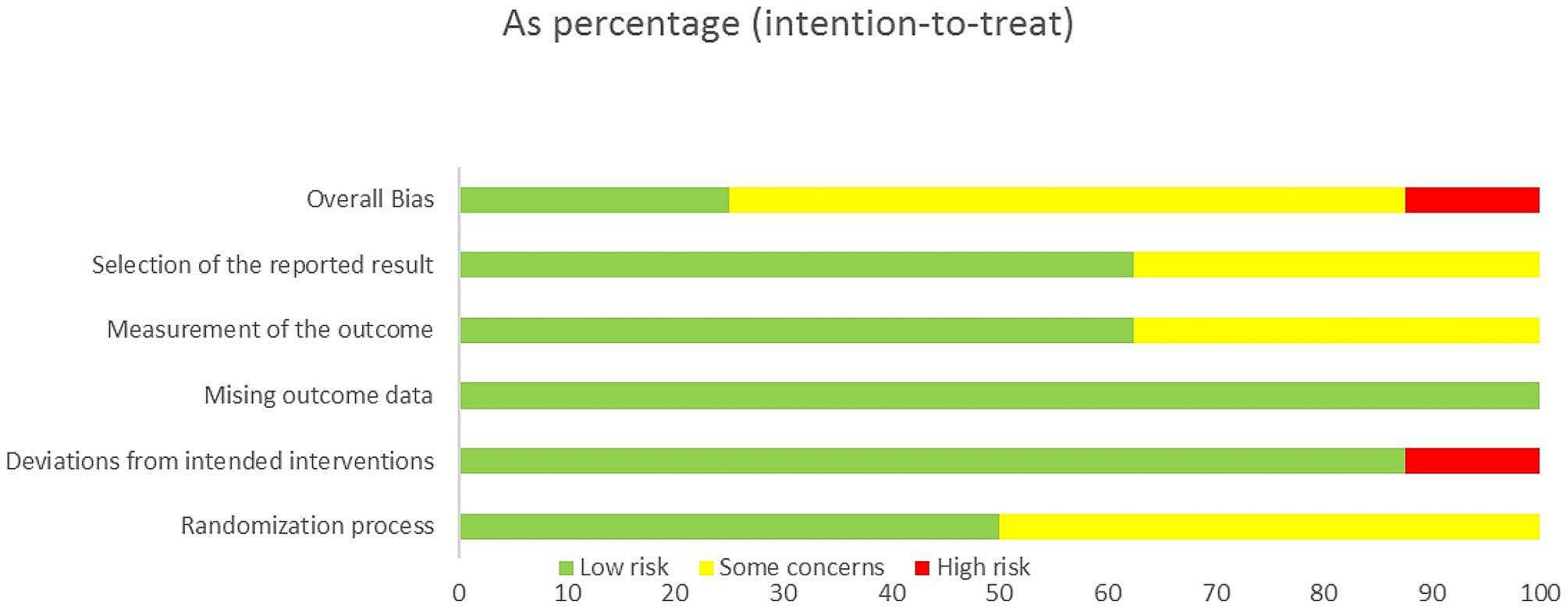
Risk of bias summary: Review authors’ judgments about each risk of bias item for each included study

Missing outcome data bias was low risk in all the studies regarding these terms. Most of the included studies [41, 44, 46–49] had a low risk of bias in the measurement outcome bias due to all outcome assessors being blinded and appropriate methods being used to measure outcomes, and 3 studies [43–45] were rated as some concern due to insufficient information regarding blinding of outcome assessors. For the selection of the reported results, five studies [45–49] were considered low risk because all measurements and analyses of the data mentioned in the results were available in the protocol. Three studies [41, 43, 44] were rated as some concern due to the lack of protocols. The overall risk was low risk for two studies [46, 49], some concern for five studies [41, 43, 45, 47, 48], and high risk for one study [44]. (Figures 2 and 3).

Using the ROBINS-1 tool, the overall risk of bias was assessed as serious in non-randomized studies [42] (Additional File, Table 2).

### Meta-analysis

#### Preoperative anxiety

The Information video before elective cesarean delivery compared to the control group may have little or no effect on preoperative anxiety, but the evidence is uncertain (SMD − 0.22, 95% CI -0.51 to 0.06, 9 trials, 1020 participants, I^2^ = 80%; very low-certainty evidence).

A subgroup analysis based on the type of intervention (information about only anesthesia versus information about anesthesia and cesarean) suggested a decrease in preoperative anxiety for information about only anesthesia (SMD − 0.28, 95% CI -0.48 to -0.07, 5 trials, 523 participants, I^2^ = 24%). There was no significant difference in this result compared to controls for information about anesthesia and cesarean (SMD − 0.18, 95% CI -0.79 to 0.44, 4 trials, 497 participants, I^2^ = 91%).

**Fig. 4:**
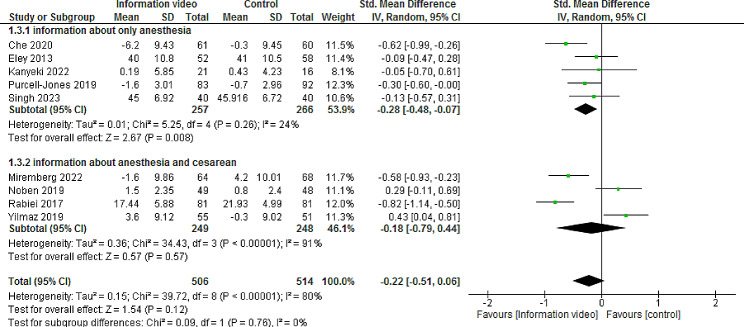
Forest plot of the difference between the mean and the standard deviation before and after of information video prior to elective cesarean delivery on preoperative anxiety (based on the type of intervention)

**Fig. 5:**
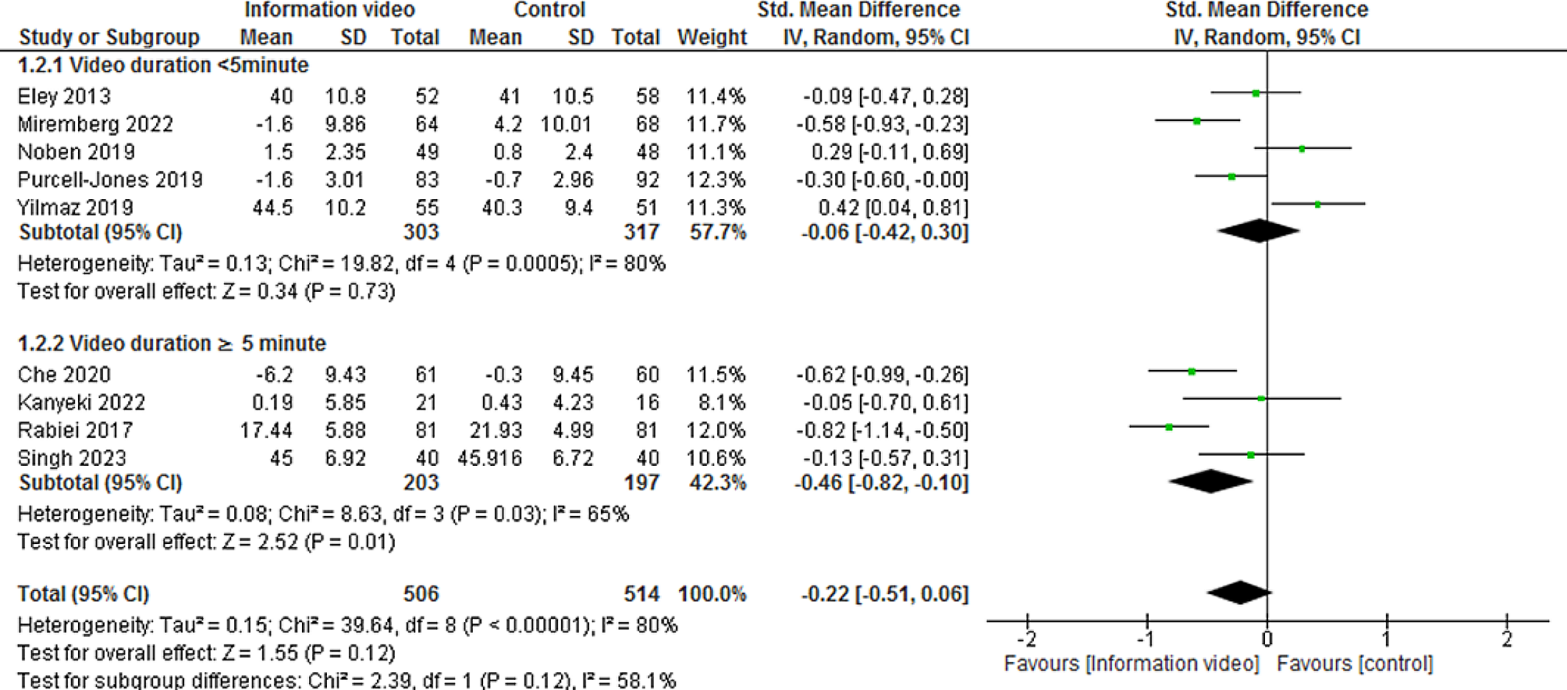
Forest plot of the difference between the mean and the standard deviation before and after of information video prior to elective cesarean delivery on preoperative anxiety (based on video duration)

A post hoc subgroup analysis based on video duration found that videos longer than 5 min may lead to a significant reduction in preoperative anxiety (SMD − 0.46, 95% CI -0.82 to -0.10, 3 trials, 400 participants, I2 = 65%). There was no significant difference in this result compared to controls for durations less than 5 min (SMD − 0.06, 95% CI -0.42 to 0.30, 5 trials, 620 participants, I^2^ = 80%) (Figs. 4 and 5).

### Operative satisfaction

**Fig. 6:**
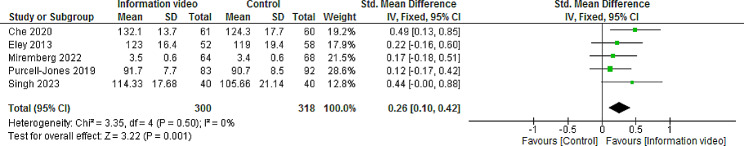
Forest plot of the effect of information video prior to elective cesarean delivery on post-operative satisfaction

The information video before elective cesarean delivery compared to the control group probably increases operative satisfaction. (SMD 0.26, 95% CI 0.10 to 0.42, 5 trials, 618 participants, I^2^ = 0%; Moderate-certainty evidence) (Fig. 6).

### Sensitivity analysis

When the non-randomized study [42] was removed from the meta-analysis, the results showed that the outcome of preoperative anxiety and operative satisfaction were not sensitive to the study type (Table 3).

**Table 3:** Sensitivity analysis of outcomes with excluding non-randomized studies

Outcomes		SMD	CI (95%)	P-value
Preoperative anxiety	Meta-analysis of all studies	− 0.22	(-0.51, 0.06)	0.12
	Sensitivity analysis	− 0.21	(-0.54, 0.12)	0.22
Post-operative satisfaction	Meta-analysis of all studies	0.26	(0.10, 0.42)	0.001
	Sensitivity analysis	0.32	(0.13, 0.51)	< 0.001

SMD: Standard Mean Difference; CI: Confidence Interval

### Certainty of evidence

The evidence for preoperative anxiety was downgraded by one level due to the risk of bias caused by detection and performance bias and by two levels for inconsistency with an I2 of 80%. Similarly, the evidence for operative satisfaction was downgraded by one level due to the risk of bias caused by detection and performance bias (Table 4).

**Table 4:** Use of the information video versus usual care

No of studies	Design	Risk of bias	Inconsistency	Indirectness	Imprecision	Publication bias	Use of the information video	Use of usual care	Pooled effect SMD (95% CI)	Final judgment
Preoperative anxiety										
9	RCT^*^ And Non-randomized	Serious	Very serious	No serious	No serious	**Suspected**	506	514	SMD 0.22 lower, (95% CI 0.51 lower to 0.06 higher)	⊕⊖⊖⊖ Very Low
**Post-operative satisfaction**										
5	RCT^*^ an Non-randomized	Serious	No serious	No serious	No Serious	**Suspected**	300	318	SMD 0.26 higher, (95% CI 0.10 higher to 0.42 higher)	⊕⊕⊕⊖ Moderate
**CI =** confidence interval; **RCT =** randomized controlled trial; **SMD =** standardized mean difference										

GRADE Working Group grades of evidence

High certainty: We are very confident that the true effect lies close to that of the estimate of the effect

Moderate certainty: We are moderately confident in the effect estimate; the true effect is likely to be close to the estimate of the effect, but there is a possibility that it is substantially different

Low certainty: Our confidence in the effect estimate is limited; the true effect may be substantially different from the estimate of the effect

Very low certainty: We have very little confidence in the effect estimate; the true effect is likely to be substantially different from the estimate of effect

### Meta-regression

The random effect meta-regression analyses indicated a significant correlation between the mean age of the intervention group (β = 0.137, P < 0.001) and the mean age of the control group (β = 0.150, P = 0.0246) with effect size. With an increase in the age of the participants, the effect size also increased. Additionally, there was no significant correlation between preoperative anxiety and the total sample size (p = 0.090), year of publication (p = 0.937), percentage of participants with above-high education (p = 0.870), baseline severity (p = 0.699), type of outcome measurement tool (p = 0. 305), percentage of nulliparous women (p = 0.870), and duration of the video (p = 0.126) (Table 5).

**Table 5:** Meta-regression analysis of variables predicting preoperative anxiety

Continuous variables	Number of studies	Regression coefficient (SE)	95% CI	p Value	Q(model)
Mean age of intervention group (Year)	9	0.137 (0.052)	0.033 to 0.241	**P < 0.001**	6.74
Mean age of control group (Year)	9	0.150 (0.067)	0.019 to 0.282	**0.0246**	5.05
Total sample size	9	-0.005 (0.003)	-0.012 to 0.0009	0.090	2.87
Baseline anxiety severity	6	-0.004 (0.011)	-0.027 to 0.018	0.699	0.15
Year of publication	9	-0.004 (0.054)	-0.110 to 0.102	0.937	0.01
High education (%)	6	0.001 (0.010)	-0.018 to 0.021	0.870	0.03
Duration of video (Minute)	9	-0.066 (0.0436)	-0.152 to 0.018	0.126	2.34
Nulliparous (%)	6	-0.011 (0.010)	-0.032 to 0.010	0.305	1.05
**Categorical variables**					
Outcome measurement tool (Reference: STAI vs. other tools)	9	-0.105 (0.324)	-0.740 to 0.530	0.746	0.10

SE: standard error; CI: confidence interval; Q: fit of model without heterogeneity; STAI: The State-Trait Anxiety Inventory. The bold values show the significant p-values

## Discussion

The present study demonstrated that watching an informational video prior to an elective cesarean section resulted in a statistically non-significant reduction in preoperative anxiety. Furthermore, it significantly enhanced postoperative satisfaction.

In the meta-analysis, high heterogeneity was observed in the outcome of preoperative anxiety. To identify the underlying cause of this high heterogeneity, subgroup analyses were conducted based on intervention content and video duration. We found that performing subgroup analysis based on information about only anesthesia versus information about anesthesia and cesarean did not result in a significant difference (P = 0.12). Similarly, post hoc subgroup analysis based on the duration of the video did not yield significant differences (P = 0.76). Additionally, the heterogeneity in the estimate points of the included studies could be attributed to various factors, such as the use of different tools to measure preoperative anxiety, methodological factors, previous maternal experiences, women’s level of awareness of the educational video, fear of maternal death, and fear of surgical complications [55].

In two studies, it was observed that the preoperative anxiety score increased following the viewing of an informational video. In Noben et al.‘s study, the intervention group experienced an increase of 1.5 points, while the control group had an increase of 0.8 points. Notably, the control group in this study had a higher level of education, and both women and their partners participated in watching the videos and providing evaluations [47]. In Yilmaz et al.‘s study, after watching the video, a significant increase in preoperative anxiety was observed in both the control and intervention groups. Unlike other included studies that focused on regional anesthesia training, the video content in this study pertained to general anesthesia, and the participant’s level of education was not mentioned [45]. Considering these findings, we included the level of education as a confounding factor in the meta-regression analysis. However, statistically, no significant difference was found between the effect size and the participant’s level of education in the included studies.

Additionally, a cross-sectional study involving 392 women in North Central Ethiopia revealed that nulliparous women experience higher levels of preoperative anxiety before elective cesarean Sect. [31]. However, the meta-regression analysis did not show a statistically significant difference between the effect size and the percentage of nulliparous women. This lack of significance may be attributed to the variations in the included study locations and the larger number of participants involved. It is worth noting that out of the nine included studies, six of them reported the number of nulliparous women in their study.

In an RCT involving 80 women, it was demonstrated that providing four training sessions on cesarean section and familiarizing women with the operating room environment resulted in a significant reduction in preoperative anxiety [56]. Furthermore, in a Scoping Review [18] that focused on preoperative educational sessions before cesarean delivery and their effect on women’s anxiety, it was shown that these sessions could lead to a reduction in anxiety among women, contrary to the findings of the current study. This study considered three types of interventions: mental health training, video (three studies), and health instruction. Out of these three relevant studies, only one study [49] met the criteria for the current systematic review and meta-analysis. The other two studies were not included because one study [57] involved an educational video about natural childbirth and women’s preference for the delivery type, and the other study [58] reported anxiety among men and women undergoing major operations, but the anxiety was not explicitly reported for cesarean delivery. Therefore, they did not meet the criteria for inclusion in the current study.

Although our meta-analysis revealed that video training can significantly enhance postoperative satisfaction, this finding was only statistically significant in Che et al.‘s study [46] among the five studies included in the present meta-analysis. In the remaining studies, the increase in postoperative satisfaction was not statistically significant [42, 44, 48, 49]. Furthermore, Yilmaz et al.‘s study [45] was not included in the meta-analysis because they reported postoperative satisfaction as a number and percentage for the intervention group compared to the control group (85% vs. 90%; p = 0.71). Therefore, it is recommended that future studies report the results in the form of mean and SD to facilitate meta-analysis.

The level of certainty in the evidence regarding preoperative anxiety was very low, highlighting the need for further studies with appropriate methodology and larger sample sizes. It is important to consider methods to minimize the risk of bias, especially in areas such as randomization, concealment of allocation, blinding, and registration of the study protocol before the start of the study.

The level of certainty in the evidence for postoperative satisfaction was moderate, suggesting that it is likely to be close to the truth. Therefore, as a psychological intervention, the use of informational videos is recommended in practice to improve satisfaction following a cesarean section. This intervention is safe, non-invasive, and does not carry any complications.

### Strengths and limitations

This study represents the first systematic review and meta-analysis examining the effect of information videos before elective cesarean delivery on preoperative anxiety and postoperative satisfaction. Additionally, we employed a comprehensive search strategy to identify relevant studies and conducted sensitivity, subgroup, and meta-regression analyses to explore potential factors influencing the results. Furthermore, we conducted an assessment of the certainty of evidence for both preoperative anxiety and post-operative satisfaction.

The present systematic review and meta-analysis have several limitations. One of the main limitations is the inclusion of studies published only in English and Persian languages, which may introduce publication bias by excluding relevant studies published in other languages or the grey literature. Additionally, due to the small number of studies available for each outcome, it was not feasible to assess publication bias. The limited number of studies and their relatively low quality could also introduce bias into the results. Furthermore, the diversity in the ethnicity of the women included in the studies may impact the generalizability of the outcomes.

## Conclusion

This systematic review and meta-analysis examined the impact of watching an informational video prior to elective cesarean delivery on preoperative anxiety. The findings suggest that watching such a video may lead to a reduction in preoperative anxiety. However, the observed reduction was not statistically significant, and there was a high degree of inconsistency across the studies included in the analysis.

Furthermore, this intervention shows potential for improving women’s postoperative satisfaction. In future studies, more research is needed to determine the optimal duration and content type of the informational video. It is advisable to consider incorporating physiological indicators such as blood pressure, heart rate, and respiration, as these factors are closely associated with anxiety levels.

### Electronic supplementary material

Below is the link to the electronic supplementary material.

**Supplementary Material 1:** Search strategy for databases of PubMed, Cochrane, Web of Science and Scopus

## Data Availability

Data obtained from the original published studies.

## Abbreviations

APAIS: Amsterdam Preoperative Anxiety and Information Scale
BMI: body mass index
CI: confidence interval
MSSCS: Maternal satisfactions scale
NVAAS: Numerical Visual Analog Anxiety Scale
RCT: Randomized controlled trial
SMD: standardized mean difference
STAI: State-Trait Anxiety Inventory
VAS: visual analog scale
