# Supplementary material for: Informational video on preoperative anxiety and postoperative satisfaction prior to elective cesarean delivery: a systematic review and meta-analysis
Source: BMC Psychol. 2024 Jan 2;12:6. doi: 10.1186/s40359-023-01499-3 (PMC10759807; doi:10.1186/s40359-023-01499-3)
Supplement: Supplementary file 1 — Supplementary Material 1: Search strategy for databases of PubMed, Cochrane, Web of Science and Scopus [file 40359_2023_1499_MOESM1_ESM.docx]

Additional file 1:

**Search Strategy**

**PubMed:**

**#1** ("controlled clinical trial"[Publication Type]) OR ("randomized trial"[Title])) OR (trial[Title])) OR (randomly[Title])) OR (randomized[Title])) OR (RCT[Title])

**#2** (cesarean[Title/Abstract])) OR ("elective caesarean"[Title/Abstract])) OR ("C-section"[Title/Abstract])) OR ("c section"[Title/Abstract])) OR ("Cesarean Section"[Title/Abstract])) OR ("Elective Surgical Procedures*"[Title/Abstract])) OR ("cesarean delivery"[Title/Abstract])) OR (childbirth [MeSH Terms])) OR (cesarean section [MeSH Terms]))

**#3** ("Informative* Video"[Title/Abstract]) OR (Media[Title/Abstract])) OR ("audio video"[Title/Abstract])) OR (multimedia[Title/Abstract])) OR ("Patient Education"[Title/Abstract])) OR (video[Title/Abstract])) OR ("Video-based information"[Title/Abstract])) OR ("virtual reality"[Title/Abstract])) OR ("preoperative information"[Title/Abstract])) OR ("Preoperative Care"[Title/Abstract])) OR (multimedia [MeSH Terms])) OR (video [MeSH Terms])

**#4** (Stress[Title/Abstract] OR Anxiety[Title/Abstract] OR Panic[Title/Abstract] OR psychology[Title/Abstract]) OR ("preoperative anxiety"[Title/Abstract])) OR ("maternal anxiety"[Title/Abstract])) OR (Anxiety [MeSH Terms])

**#5** (satisfaction[Title/Abstract]) OR (Patient satisfaction[Title/Abstract])) OR ("operative satisfaction"[Title/Abstract])) OR ("Surgical satisfaction"[Title/Abstract])) ) OR (patient satisfaction[MeSH Terms])

**#6** #4 OR #5

**#7** #1 AND #2 AND #3 AND #6

***Cochrane:***

**#1** MeSH descriptor: [Multimedia] explode all trees

**#2** video OR "media" OR "audio video" OR "virtual reality" OR "Informative* Video" OR "Preoperative Care" OR "preoperative information" OR "Information Video" OR "Video-based information"

**#3** #1 OR #2

**#4** cesarean OR "elective caesarean" OR "C-section" OR "c section" OR "Cesarean Section" OR "Elective Surgical Procedures*" OR "cesarean delivery"

**#5** MeSH descriptor: [Parturition] explode all trees

**#6** MeSH descriptor: [Cesarean Section] explode all trees

**#7** #4 OR #5 OR #6

**#8** satisfaction OR Patient satisfaction OR "operative satisfaction" OR "Surgical satisfaction"

**#9** MeSH descriptor: [Patient Satisfaction] explode all trees

**#10** #8 OR #9

**#11** Stress OR Anxiety OR Panic OR psychology OR "preoperative anxiety" OR "maternal anxiety"

**#12** MeSH descriptor: [Anxiety] explode all trees

**#13** #11 OR #12

#**14** #10 OR #13

**#15** "controlled clinical trial" OR "randomized" OR "randomly" OR "trial" OR RCT

**#16** #14 AND #7 AND #3

**Web of Science:**

**#1** ("controlled clinical trial" OR "randomized" OR "randomly" OR "trial" OR RCT) (Topic)

**#2** TS= video OR "media" OR "audio video" OR "virtual reality" OR "Informative* Video" OR "Preoperative Care" OR "preoperative information" OR "Information Video" OR "Video-based information"

**#3** TS= (cesarean OR "elective caesarean" OR "C-section" OR "c section" OR "Cesarean Section" OR "Elective Surgical Procedures*" OR "cesarean delivery")

**#4** TS= (satisfaction OR Patient satisfaction OR "operative satisfaction" OR "Surgical satisfaction")

**#5** TS= (Stress OR Anxiety OR Panic OR psychology OR "preoperative anxiety" OR "maternal anxiety")

**#6** #4 OR #5

**#7** #1 AND #2 AND #3 AND #6

**Scopus**

**1#** TITLE-ABS-KEY (video ) OR TITLE-ABS-KEY ( "media" ) OR TITLE-ABS-KEY ( "audio video" ) OR TITLE-ABS-KEY ( "virtual reality" ) OR TITLE-ABS-KEY ( "Informative* Video" ) OR TITLE-ABS-KEY ( "Preoperative Care" ) OR TITLE-ABS-KEY ( "preoperative information" ) OR TITLE-ABS-KEY ( "Information Video" ) OR TITLE-ABS-KEY ( "Video-based information" ) )

**#2** TITLE-ABS-KEY (cesarean) OR TITLE-ABS-KEY ("elective caesarean") OR TITLE-ABS-KEY ("C-section" ) OR TITLE-ABS-KEY ( "c section" ) OR TITLE-ABS-KEY ( "Cesarean Section" ) OR TITLE-ABS-KEY ( "Elective Surgical Procedures*" ) OR TITLE-ABS-KEY ( "cesarean delivery" ) )

**#3** TITLE-ABS-KEY (satisfaction) OR TITLE-ABS-KEY (patient AND satisfaction) OR TITLE-ABS-KEY ("operative satisfaction") OR TITLE-ABS-KEY ("Surgical satisfaction")

**#4** TITLE-ABS-KEY (stress) OR TITLE-ABS-KEY (anxiety) OR TITLE-ABS-KEY (panic) OR TITLE-ABS-KEY (psychology) OR TITLE-ABS-KEY ("preoperative anxiety") OR TITLE-ABS-KEY ("maternal anxiety"))

**#5** #3 OR #4

**#6** TITLE-ABS-KEY ( "controlled clinical trial" OR randomized OR randomly OR trial OR rct ) )

**#7** #1 AND #2 AND #5 AND #6

**Table 1** Risk of bias in quasi-randomized clinical trials according to ROBINS-I.

| **Author** | **Purcell-Jones et al**  **2019** |
| --- | --- |
| Bias due to confounding | Low |
| Bias in selection of participants | Serious |
| Bias in the classification of interventions | Low |
| Bias due to deviations from intended interventions | Low |
| Bias due to missing data | Serious |
| Bias in measurement of outcomes | moderate |
| Bias in selection of reported result | Low |
| Overall | Serious |

Low: low risk of bias [the study is comparable to a well-performed randomized trial with regard to this domain); No information: no information on which to base a judgment about risk of bias for this domain; Serious: serious risk of bias [the study has some important problems

**Table 2** The mean and standard deviation for preoperative anxiety scores before and after the intervention

| **First**  **Author/ date of publication** | **Before the intervention** | | **After the intervention** | |
| --- | --- | --- | --- | --- |
|  | **Intervention group** | **Control group** | **Intervention group** | **Control group** |
| Kanyeki et al.  2022 | 46.86 ± 5.34 | 45.63 ± 4.35 | 47.05± 6.26 | 46.06 ± 4.11 |
| Eley et al.  2013 | NR | NR | 40.0 ± 10.8 | 41±10.5 |
| Miremberg et al.  2022 | 42.9 ±10.2 | 45.1±9.7 | 41.3±9.5 | 49.3±10.3 |
| Noben et al.  2019 | 4.1±2.3 | 3.8±2.3 | 5.6±2.4 | 4.6±2.5 |
| Che et al.  2020 | 36.8±10.4 | 36.8±9.4 | 30.6±8.0 | 36.5±9.5 |
| Yilmaz et al.  2019 | 40.9±7.4 | 40.6±8.6 | 44.5±10.2 | 40.3±9.4 |
| Purcell-Jones et al.  2019 | 4.2±3.2 | 4.0±3.1 | 2.6±2.8 | 3.3±2.8 |
| Rabiei et al.  2017 | NR | NR | 17.44±5.88 | 21.93±4.99 |
| Singh et al.  2023 | NR | NR | 45±6.92 | 45.91±6.72 |
